# Attitudes in an interpersonal context: Psychological safety as a route to attitude change

**DOI:** 10.3389/fpsyg.2022.932413

**Published:** 2022-07-26

**Authors:** Guy Itzchakov, Kenneth G. DeMarree

**Affiliations:** ^1^Department of Human Services, University of Haifa, Haifa, Israel; ^2^Department of Psychology, University at Buffalo, Buffalo, NY, United States

**Keywords:** psychological safety, interpersonal interactions, self presentation, attitude change, persuasion, attitude strength

## Abstract

Interpersonal contexts can be complex because they can involve two or more people who are interdependent, each of whom is pursuing both individual and shared goals. Interactions consist of individual and joint behaviors that evolve dynamically over time. Interactions are likely to affect people’s attitudes because the interpersonal context gives conversation partners a great deal of opportunity to intentionally or unintentionally influence each other. However, despite the importance of attitudes and attitude change in interpersonal interactions, this topic remains understudied. To shed light on the importance of this topic. We briefly review the features of interpersonal contexts and build a case that understanding people’s sense of psychological safety is key to understanding interpersonal influences on people’s attitudes. Specifically, feeling psychologically safe can make individuals more open-minded, increase reflective introspection, and decrease defensive processing. Psychological safety impacts how individuals think, make sense of their social world, and process attitude-relevant information. These processes can result in attitude change, even without any attempt at persuasion. We review the literature on interpersonal threats, receiving psychological safety, providing psychological safety, and interpersonal dynamics. We then detail the shortcomings of current approaches, highlight unanswered questions, and suggest avenues for future research that can contribute in developing this field.

## Introduction

Carla had a tough day at work. She had a vast number of things to do and could not get all of them done. Carla feels her manager puts too much pressure on her and is thinking about quitting her job. After work, she meets her friend Cheryl at their favorite coffee house and tells her all about it. Cheryl listens to Carla attentively, gives her time and space to speak her mind, helps clarify the problem, and asks questions that show understanding. As Carla sips her coffee and tells her story, she recalls another instance when she was under even more pressure at work but had no problem dealing with it because she enjoyed her job. As the conversation continues, Carla realizes the main problem is not her manager but rather her drop in motivation.

Although people engage in this type of interaction on a daily basis, interactions are far from straightforward. Individual participants have their own goals (Carla wants to vent her frustration, Cheryl wants to support her friend) as well as shared goals (to develop and maintain their friendship and enjoy a night out). These interactions are dynamic over time and involve conversational turn-taking, where each participant responds to the other’s verbal and non-verbal behaviors and provides opportunities for mutual influence. The idea that such interactions can influence both parties is the focus of the present study. Although many factors can determine the amount and direction of influence in interpersonal interactions, this review centers on one construct we suggest is central to understanding influence in an interpersonal context: psychological safety. Below, we explore the role of psychological safety from three perspectives: (a) reducing self-threat and uncertainty, (b) how and why *receiving* psychological safety from others can change the attitudes of an interactant, and (c) how *providing* psychological safety to others can change the attitudes of the providers. Then, we discuss attitude change in the context of interpersonal dynamics, followed by a discussion of the limitations and unanswered questions in this research area, and provide suggestions for future research.

In the empirical literature, the dominant approach to studying attitude change is to present participants with a persuasive message such as an ostensible advertisement, newspaper editorial, or political speech, and then measure their responses to this stimulus (Maio et al., 2018). By manipulating or measuring aspects of the recipients’ preexisting beliefs, attitudes, or mindsets (e.g., initial attitude, expectations, mood), aspects of the source (e.g., expertise or attractiveness), aspects of the message itself (e.g., strength of the arguments or types of arguments used), and various combinations of the above, much has been learned about processes underlying attitude change. This approach has led to contemporary models of persuasion such as the Elaboration Likelihood Model (ELM; Petty and Cacioppo, 1986) and the Heuristic-Systematic Model (Chaiken et al., 1989) that have decades of empirical support. Despite the many strengths of the dominant approach, it largely ignores an essential context for persuasion: interpersonal interactions.

Interpersonal interactions present a novel context for studies on attitude change. The dynamic nature of interactions means that any of the parties to the interaction can be the source or the recipient of a message at any given time. In addition, rather than only internally processing a fixed message, in an interpersonal context, recipients can overtly express their agreement or disagreement directly to the source, counterargue specific points, ask questions of the source, or any of a range of other verbal or non-verbal responses (e.g., nodding, changing the topic, looking at their phone, etc.). Recipient responses can then affect the source, resulting in a change in behavior, including persuasive strategies, or their own attitudes. A full review of all of these factors is beyond the scope of this paper. Instead, we focus on psychological threat and safety in interpersonal interactions.

A psychological threat involves the perception that some aspect of the self (e.g., one’s freedom, inclusion, health, or importantly held views) is in jeopardy. Threats can take many forms in interpersonal interactions. These can include the possibility that one’s inclusion is in jeopardy, which can come from very clear cues of rejection or ostracism, or from uncertainty or ambiguity about one’s inclusion in the interaction (e.g., prior to an interaction with a new person, one’s potential for inclusion is unknown; Han et al., 2015). Threats can also come from information conveyed in an interaction that negatively implicates oneself (e.g., messages indicating that one’s existing behavior is unhealthy; Sherman et al., 2000) or directly attacking one’s views or autonomy, e.g., when an interaction partner explicitly tries to persuade, this threatens the attitude under attack as well as one’s freedom to hold it (Rosenberg and Siegel, 2021). Threats like these can undermine psychological safety.

Psychological safety is often defined as the perception that it is safe to voice one’s opinion and take interpersonal risks without fear that such risks will backfire (Edmondson and Lei, 2014). In a psychologically safe environment, people feel they will not be rejected for being authentic and expressing their thoughts. This type of atmosphere involves the parties’ positive intentions toward one another and engaging in constructive disagreements (Edmondson, 1999). However, we conceptualize safety more broadly to include a sense of acceptance and inclusion by one’s interaction partners. We build on Carl Rogers’ notion of an atmosphere of safety (Rogers, 1980). According to Rogers, speakers feel safe expressing themselves authentically without fear of being judged by their listeners in an atmosphere of safety. There are many ways that interactions with others can undermine psychological safety, for example when people want specific outcomes such as getting others to accept their proposal. The dependence of these outcomes on the interaction partner(s) means that these outcomes will be contingent on the other person and thus are uncertain. However, when a person’s interaction partner tries to force them to change their attitude, the persuasion attempt may threaten their sense of freedom and autonomy (Brehm and Brehm, 1981). These uncertainties or threats undermine the person’s psychological safety, leading them to experience a state of anxiety. The potential threat people experience in these interactions can lead to a range of self-presentational attempts and defensive responses (Kunda, 1990).

Although threat and psychological safety are related in many cases, we do not perceive them as two ends of a continuum. As noted above, threats in an interpersonal context can take many forms, including threats to inclusion, threats to autonomy, and threats to one’s attitudes or beliefs. We view psychological safety as promoting inclusion and acceptance, and a person’s inherent value. In other words, a person may be in an interaction that is psychologically safe but which may still threaten an attitude or a belief held by that person. Furthermore, the absence of a threat in the interaction does not necessarily mean the presence of psychological safety, such as when a person is not at risk of being rejected but is also not fully included or accepted in an interaction. Put differently, a threat can co-occur with psychological safety. In an atmosphere of safety, tough conversations can take place, including ones that challenge important views, thus often eliciting feelings of threat. However, when people experience psychological safety, they feel understood and accepted and maintain their autonomy, even when disagreements and conflict are part of the interaction.

This definition of psychological safety intersects with the desire to feel understood and appreciated during an interaction (Reis and Gable, 2003), which is different from having one’s attitude verified (cf. Swann, 1990). Feeling understood promotes psychological safety (Itzchakov et al., 2022a). Individuals can feel understood in an interaction even when their attitudes and values are not verified (Reis et al., 2017). For example, romantic partners in a happy relationship can have their attitudes on a topic threatened and challenged while still maintaining psychological safety during the disagreement (but see; Tynan, 2005). In addition, feeling understood in a romantic relationship helps prevent the adverse consequences of interpersonal conflicts (Gordon and Chen, 2016).

Interaction partners who provide their partners with psychological safety free them from concerns of rejection and judgment. This allows for speakers to introspect on their attitudes in a non-defensive manner, which can lead to a broader and more complex perspective on the topics under discussion (Rogers, 1980). Providing psychological safety helps its recipients by, for example, increasing their vitality (Kark and Carmeli, 2009) and creativity (Carmeli et al., 2010). Providing psychological safety does not mean that interactants necessarily agree with each other, but rather that they acknowledge each other’s autonomy and inherent value.

How do people communicate psychological safety? A variety of behaviors has been suggested. A prominent behavior is high-quality listening. High-quality listening is defined as being attentive, responsive, and non-judgmental to one’s interaction partner, demonstrating curiosity and attempting to understand one’s partner, and validating one’s partner’s point of, even when expressing disagreement (Kluger and Itzchakov, 2022). Psychological safety can also be achieved in a difficult conversation between long-term romantic partners or close friends when the partner demonstrates unconditional acceptance (Waldron and Kelley, 2005) or when individuals feel that their partner is responsive to their needs, desires, and core values (Reis et al., 2004).

As should be clear from this discussion, psychological safety and interpersonal threats are related. The more an interaction partner feels psychologically safe to self-express, the less interpersonal threat this individual will perceive. Nevertheless, the two constructs are not simply two ends of one continuum. Although safety is more likely in the absence of threats and vice-versa, the absence of threats does not guarantee psychological safety. Furthermore, as many of the studies reviewed below demonstrate, a sense of psychological safety can coexist with conditions that normally elicit threats. This observation is one of the key reasons for studying psychological safety. For example, a sense of psychological safety in work teams leads people to be more likely to be open to colleagues who challenge their views, which would typically induce a sense of threat (Argyris and Schon, 1978; Edmondson, 1999).

We organize our review into five sections. The first section describes how interpersonal interactions can pose threats, such as by threatening belonging needs or one’s attitudes, and describes how people respond to these potential threats. The second section describes how the experience of psychological safety can influence one’s attitudes in attitude-relevant conversations; that is, how and why attitudes change when speakers feel that they can share their message without being judged or criticized by the recipients. The third section describes situations where the communicator of a message provides psychological safety to the recipient. The fourth section describes studies on interpersonal dynamics with potential implications for attitude change. Finally, we discuss the limitations and open questions in the literature and suggest avenues for future research. Our review is necessarily limited, but we attempted to be as thorough as possible by searching key databases (Psychinfo, Web Of Science, and Google Scholar) with multiple combinations of keywords (e.g., interpersonal persuasion, interpersonal influence, etc.), in addition to the research we were already aware of.

## Interpersonal threats

Individuals are fundamentally motivated to feel that they belong in social settings (Baumeister and Leary, 1995; Leary and Gabriel, 2022). This need to belong motivates people to seek connection, inclusion, and positive regard from others. Fear of an upcoming interaction can threaten one’s need to belong, especially when the relationship is not firmly or securely established. This can have a range of implications for people’s attitudes. For example, when interacting with a new person, recruiting attitudes that might help the person “fit in” is one potential self-presentational strategy since attitudinal similarity predicts interpersonal liking (Byrne, 1961) even outside of laboratory settings (Montoya et al., 2008). Consistent with this strategy, Cialdini et al. (1973) found that when people anticipated interacting with someone whose opinions differed from their own, they shifted their opinions toward their prospective partner’s attitudes. A study by Tetlock et al. (1989) suggests that this strategy tends to be adopted when one is not pre-committed to a particular viewpoint and one’s interaction partner’s attitudes are known.

The motivation to fit in and have a smooth interaction can be particularly strong when a member of an advantaged group interacts with a member of a stigmatized group. Members of advantaged groups often worry about misbehaving in interactions with members of disadvantaged groups (Shelton et al., 2005b), thus increasing the potential psychological threat of intergroup interactions. Norton et al. (2012) argued that these concerns might elicit agreeable responses and make it particularly difficult to decline a request from a member of a stigmatized group. For example, in study 2, White participants received a persuasive message from a Black or a White confederate either in person (where these motives would be activated) or on video (where they would not be). Participants were only found to be more persuaded by a Black than a White confederate in the in-person conditions. Thus, concerns about having a smooth interaction and avoiding rejection can lead people to endorse attitudes shared by an interaction partner.

However, shifting one’s attitude to coincide with those of an interaction partner is not an effective way to “fit in” when one’s interaction partner’s attitudes are unknown. Cultivating flexibility may be more beneficial to achieving acceptance in such situations, because people will be ready to respond appropriately regardless of their partner’s attitudes. Consistent with this idea, Tetlock et al. (1989) found that people engage in a more complex and integrative thought when the attitudes of a prospective interaction partner are unknown and there is no preexisting commitment to a particular viewpoint. Pillaud et al. (2013) provided supportive evidence that people cultivate ambivalence on controversial issues when pursuing self-presentational motives. In such contexts, ambivalence might communicate thoughtfulness and competence (Pillaud et al., 2018) and give a person flexibility to agree, at least in part, with aspects of the partner’s attitudes. Thus, these outcomes in the form of more complex thinking and ambivalence may give a person greater flexibility to find a way to be included and avoid possible social rejection.

Concerns about fitting in are not the only potential threat in interpersonal interactions. In particular, if an interaction partner *disagrees*, the disagreement itself might be a threat, at least if the attitude is personally meaningful. Under these conditions, defending one’s attitude may have higher motivational priority than concerns over inclusion. This notion has been supported in various findings in which participants responded to disagreements over important or committed opinions by bolstering their preexisting views and becoming more extreme (e.g., Cialdini et al., 1976; Tetlock et al., 1989).

It is worth noting that research has examined other strategies people use to prepare for interactions, which can vary as a function of their interaction goals. For example, knowing that they have to share the reasons for consumer preferences in an interaction might lead a person to rehearse their main points prior to the conversation (Schlosser and Shavitt, 1999). This strategy could enable the interaction to proceed more smoothly, but the situational activation of a subset of attitude-relevant knowledge can also shift people’s attitudes (e.g., Schwarz, 2007). Critically, Schlosser and Shavitt (2002) found that rehearsal can lead to moderation, polarization, or no change in people’s attitudes depending on evaluative connotations of the rehearsed information.

Some of the studies discussed in this section raise questions as to how the desire to fit in *actually* influences people’s attitudes. For example, when people shift their attitudes toward those of a potential interaction partner, the observed change may merely represent a shift in people’s *reports* of their attitudes but not their internally endorsed attitudes. In other cases, such as when people selectively activate particular aspects of attitude-relevant knowledge (e.g., by rehearsal), the shifts in attitudes may reflect actual changes in how people evaluate the target object, at least at the moment. However, even when people are only using self-presentational strategies, the roles they play can still become internalized (Jones et al., 1981). These internalized shifts can occur through a range of psychological processes(e.g., selective accessibility, dissonance reduction; Rhodewalt and Agustsdottir, 1986).

Ironically, a threat to an important attitude can actually promote interpersonal attitude change in some situations, such as *via* an influence technique known as paradoxical thinking. Paradoxical thinking is an attempt to change a person’s attitude by providing consistent yet amplified and exaggerated messages to the recipient’s initial point of view. The goal of paradoxical thinking is to make attitude holders perceive that their initial attitude is irrational (Hameiri et al., 2014). This appears to be particularly effective in individuals who already have extreme views as long as the amplified position is not too extreme (Hameiri et al., 2018). An important driver of paradoxical thinking is that recipients feel their identity has been threatened (Hameiri et al., 2018). Thus, by increasing the self-threat using extreme questions or statements, conversation partners can moderate the extent to which their speakers hold extreme attitudes (Bar-Tal et al., 2021).

As noted, a disagreement on importantly held attitudes is potentially threatening and may lead people to double down on their preexisting views. This will generally have negative implications for interactions (Frimer and Skitka, 2020), because, as described below, high-quality interactions often flourish in the presence of curiosity and receptivity to another person’s point of view.

## Receiving psychological safety

Interaction partners can receive feedback conveying psychological safety from their partners in various ways in interpersonal interactions. For example, when listeners are verbally or non-verbally engaged, ask questions, and attempt to understand the speakers’ views, perspectives, and experiences, the speakers feel psychologically safe to share their experiences authentically (e.g., Van Quaquebeke and Felps, 2018; Weinstein et al., 2022). This psychological safety can, in turn, reduce anxiety and defensiveness and promote open-mindedness, self-reflection, and humility. In discussing one’s attitudes, we should note that a communicator could receive psychological safety from their audience or provide it to them. We discuss both situations below, beginning with situations where the *audience* provides a speaker with psychological safety.

### As the message source

The most extensive research on audience provision of a signal of psychological safety is the research by the first author and his colleagues on high-quality listening (Castro et al., 2016; Itzchakov et al., 2016, 2018a,^2020^). High-quality listening occurs when an interaction partner shows engagement and genuine interest in the speaker’s perspective and attempts to understand it (Kluger and Itzchakov, 2022). In a typical experimental paradigm, individual participants are asked to speak about their attitude toward a selected topic while interacting with another person (e.g., a confederate or another participant) whose non-verbal and verbal behaviors vary in terms of the quality of listening provided (e.g., average or moderate-quality listening vs. high-quality listening). Although these studies are generally conducted in person, sometimes as part of formal listening training, comparable effects have been obtained with written scenarios and video chat paradigms (e.g., Berkovich and Eyal, 2018). Note that high-quality listening does not mean that speakers perceive that their listeners agree with their attitudes. Rather, they should feel that they are free to express themselves without being judged or evaluated. Furthermore, listeners, through verbal (e.g., question-asking, reflection) and non-verbal behaviors (e.g., posture, eye contact), help speakers gain more self-insight and can often foster greater complexity in their attitudes.

The findings show that speakers who feel they have received high-quality listening (vs. more typical or even distracted listening) are less defensive (Itzchakov et al., 2017), feel less self-presentational concerns (Itzchakov et al., 2018a), believe they have gained more insights into their attitudes (Itzchakov et al., 2020), and feel a higher sense of relatedness toward their listeners (Weinstein et al., 2021). The implications for speakers’ attitudes are interesting. High-quality listening leads speakers to think more deeply and self-reflectively about their attitudes, resulting in changes in how they think about the topic. Speakers who feel they have received high-quality listening reflect on both sides of an issue report higher objective ambivalence (seeing the issues as having both positive and negative components) and less extreme attitudes (Itzchakov et al., 2017).

The presence of objective ambivalence typically leads people to *feel* ambivalent (subjective ambivalence; Priester and Petty, 1996), but the sense of acceptance by the listeners in these studies was shown to actually lead to a decrease in subjective ambivalence and a decoupling of the typical relationship between objective and subjective experiences of conflict (i.e., people did not necessarily feel conflicted when they hold opposing evaluations). Furthermore, the additional reflection on their attitudes may lead speakers who are listened to well to have a clearer sense of what their attitudes are (Itzchakov et al., 2018a). Attitude clarity is one form of attitude certainty (Petrocelli et al., 2007), one of the best-studied predictors of “attitude strength” (see Luttrell and Sawicki, 2020). Attitude clarity increases the strength of an attitude (Petrocelli et al., 2007) and people’s willingness to share their attitudes with others, but, unlike forms of certainty in which a people think that their viewpoint is the correct one, not attempts to persuade others or forcefully promote their attitudes (Rios et al., 2014). Thus, higher levels of attitude clarity, which can emerge when people receive high-quality listening, may increase the utility of an attitude for the attitude holder without leading to potential negative social consequences of some strongly held attitudes.

In the above cases, people did not change the valence of their attitudes, even though the structural properties or metacognitive perceptions of them changed in response to receiving high-quality listening. Prejudice is a prime example of an attitude that listening can change. People’s prejudices often reflect defensiveness (Stone et al., 2011) and entrenched views that people are unwilling to reconsider. A sense of psychological safety when talking about one’s outgroup attitudes may lead people to let down these defenses and reconsider their evaluations. In a series of studies, receiving high-quality listening when talking about one’s negative outgroup evaluations lead to a moderation of these evaluations (Itzchakov et al., 2020).

Relatedly, Voelkel et al. (2021) tested the role of political inclusion in prejudice reduction during conversations. Inclusion refers to the extent to which speakers feel free to voice their opinions, and is associated with psychological safety (e.g., Sherf et al., 2021). Speakers who felt a sense of inclusion from listeners who belonged to an outgroup reported lower prejudice toward the outgroup than speakers under control conditions (Voelkel et al., 2021). These effects were found using an imagined conversation and computer–mediated interactions. Although it is difficult to generalize beyond the specific groups examined in this study, this finding is consistent with the idea that receiving psychological safety from a member of an outgroup can shift a person’s attitudes toward that outgroup in general.

Attitude change can also be fostered in the presence of perceived responsiveness. Perceived responsiveness emerges when individuals feel that others are respectful, encouraging, and supportive (Reis, 2012). Reis et al. (2018) found that when individuals feel that others are responsive toward them, they exhibit less ego-defensiveness and higher levels of intellectual humility and can recognize that their beliefs might be wrong (Leary et al., 2017). Moreover, perceived responsiveness was shown to increase objective attitude ambivalence and intentions to act in an open-minded manner (Itzchakov and Reis, 2021). Although perceived responsiveness and listening are forms of social support that share several similarities, they are not isomorphic. For example, listening requires a conversation, whereas perceived responsiveness can be conveyed without a conversation, such as through giving a present or a hug (Itzchakov et al., 2022a).

Psychotherapy research has also examined the importance of psychological safety during conversations in attitude change. Specifically, motivational interviewing is often deployed to help clients explore their minds and attitudes. During a motivational interview, the listener, usually a licensed psychologist, a social worker, or other professional, focuses on understanding the client’s point of view by reflective listening that brings up the client’s concerns (Miller and Rose, 2009), which helps make clients aware of their ambivalence (Miller and Rose, 2015). The safe atmosphere that the listener provides during motivational interviewing enables clients to explore their ambivalence with less resistance, making attitude change possible. For example, a meta-analysis found a moderate effect of motivational interviews on adolescents’ attitudes toward using drugs (*d* = 0.44, 95% *CI* = [0.2;0.67]) (Li et al., 2016).

### As the message recipient

There are many ways that communicators’ attitudes can be changed when they are the *recipient* of psychological safety. However, the situation can sometimes be more complex when the communicator *provides* psychological safety to a message recipient. A persuasive message itself can be a potential threat to a recipient, because it can attack a personally important viewpoint or it can threaten individuals’ sense of autonomy. As we describe below, message sources that validate their audiences’ attitudinal autonomy are likely to achieve better outcomes, because this reduces the recipients’ perceptions of threat while also supporting their psychological safety.

Kalla and Broockman (2020) examined the conditions under which face-to-face canvassers can effectively produce attitude change. For example, they examined attitudes and policy preferences toward transgender people’s rights. Their intervention was multifaceted, but some features promoted a sense of understanding and psychological safety between the canvasser and the participant, such as the non-judgmental sharing of personal narratives. Their intervention successfully changed attitudes toward transgender people for at least 3 months post-intervention. Although the multifaceted nature of their intervention makes it harder to draw firm conclusions about the conditions for change, the provision of psychological safety to the audience appears to be one of the key ingredients for its success.

Studies have also examined interpersonal behaviors that communicators engage in that can affect the openness and receptivity of their audience (Minson and Chen, 2021). For example, communicators who express interest in learning about their recipients’ viewpoints elicited more open-minded (or less defensive) responses from their audiences (Chen et al., 2010). Although not typically enacted in interpersonal paradigms, evidence indicates that other behaviors by communicators that signal their own receptivity to their audience’s viewpoint, such as delivering a two-sided message (Xu and Petty, 2021), can similarly impact the receptivity of their audience (see Hussein and Tormala, 2021). Such cues to receptivity by a communicator may signal to the recipients that their viewpoints are valid to have and express even if the communicator disagrees with them. This should support audiences’ autonomy and may further convey acceptance and psychological safety, because the recipients of a receptive communicator may feel that they will not be socially rejected because of their attitudes (see Itzchakov et al., 2022b).

Receiving psychological safety is also crucial for an attitude change in group discussions. When individuals lack psychological safety, they are less likely to express their attitudes (O’Donovan and McAuliffe, 2020). When individuals feel safe expressing themselves, discussions encompass a broader range of attitudes shared, opening an avenue for attitude change. One form of group discussion that can promote psychological safety and, hence, attitude change is listening circles (or council; Zimmerman and Coyle, 2009). A listening circle is structured to shift a discussion from being unstructured and opinionated to a receptive process of speaking and listening. In a listening circle, there is structured turn-taking where those who are not speaking are instructed to listen in an open-minded and non-judgmental manner. Listening circles can enable individuals to resolve their differences by increasing the psychological safety of group members (Zimmerman and Coyle, 2009). Three field quasi-experiments compared the effects of listening circle workshops to alternative workshops. Employees who attended the listening circle workshop reported less social anxiety (similar to higher psychological safety), leading to higher objective attitude ambivalence and lower attitude extremity toward the work-related attitudes that they discussed in the listening circles (Itzchakov and Kluger, 2017).

### Parallels to self-affirmation

According to Self-Affirmation Theory, individuals become defensive when faced with information that threatens their self-views as good and moral people who act according to social norms (i.e., self-integrity; Sherman and Cohen, 2006). Individuals restore their self-integrity by promoting values, beliefs, and roles that are central to their identity (Sherman and Cohen, 2002). When individuals’ self-integrity is affirmed, they gain psychological safety (Lepper and Woolverton, 2002).

Self-affirmation is a possible exception to the general lack of research on psychological safety in persuasion (e.g., Sherman et al., 2000; Briñol et al., 2007). In self-affirmation research, an essential aspect of the participants’ self is affirmed (e.g., by writing about a personally important value), after which they are presented with an unrelated but potentially threatening persuasive message (e.g., about health risks). Research generally finds that self-affirmation reduces defensive responses and increases attitude change (e.g., Epton et al., 2015). Although self-affirmation generally occurs outside of an interpersonal context, it may provide some similar benefits or operate *via* some mechanisms that are similar to psychological safety. As with psychological safety, self-affirmation is hypothesized to bolster one’s sense of inherent worth (Steele, 1988), reduce defensive responding (Sherman and Cohen, 2006), and broaden one’s view on the topic under discussion (Critcher and Dunning, 2015). It is possible that receiving psychological safety in an interaction affirms one’s inherent social value.

### Perceived consensus

Another interesting variable to consider is the sense of psychological safety communicated when individuals’ interaction partners agree with them. Agreement on the part of many people is typically referred to as *consensus*. Such agreement validates an individual’s viewpoint and reduces concerns that their position will be challenged and changed. Petrocelli et al. (2007) found that when participants were provided with (bogus) information that the majority of other participants shared their attitude, they reported higher levels of attitude *correctness.* This validation, in turn, can lead people to engage in more advocacy (Cheatham and Tormala, 2015) but also attempt to force their attitude on others (Rios et al., 2014). Prislin et al. (2011) examined shifts in group consensus with speaker’s viewpoints in an in-person group context and found that if a group shifted its opinions to align with the speaker’s, the speaker became more certain.^1^ This shift toward consensus also prompted speakers to change their verbal and non-verbal behaviors in ways perceived to be more persuasive.

According to Prislin et al. (2000), Prislin and Christensen (2002), shifts in group consensus like those just described have a range of implications for both group processes and attitudes. For example, when group members change their attitude from the initial majority position to a minority position, they experience decreases in their perceptions of similarity with the group, lowered expectations for positive conversations, and increased expectations for negative interactions (Prislin et al., 2000; Prislin and Christensen, 2002). In other words, a shift away from consensus seems to threaten people’s belongingness needs. In contrast, when group members change their attitude from a minority position to a majority position, their tolerance for the opposite attitude decreases (Prislin et al., 2000; Prislin and Christensen, 2002), suggesting that in such situations, group members are less likely to provide individuals who have dissenting viewpoints with psychological safety.

However, it is worth considering whether consensus information actually provides psychological safety. In one sense, consensus protects people’s attitudes from a threat to self-worth by providing some external validation for the legitimacy of their viewpoint. On the other hand, although the focal attitude and its relationship to the self may be “safe,” an interaction in which a person’s sense of inclusion is contingent upon having a particular attitude (shared or unshared with the group) may not provide psychological safety. This situation might even lead to more narrow-minded thinking such as self-censorship (Janis, 1972) and rejection of divergent views.

## Providing psychological safety

The previous section reviewed studies on the effect of receiving psychological safety on attitude change. In an interpersonal context, when one receives psychological safety, one’s interaction partner (or partners) provides psychological safety. Providing psychological safety is characterized by behaviors such as engaging in high-quality listening, showing empathy, and providing emotional support. An important construct in this context is perspective-taking, which involves imagining how another person would feel, which offers the potential to increase others’ sense of psychological safety (Cho, 2022).

### Perspective-taking

When people take the perspective of others, they have the potential to provide them with psychological safety. Contrary to the research on listening and attitudes that has so far focused on attitudes of the psychological safety *receiver* (i.e., the speaker), perspective-taking research often examines attitudes of the psychological safety *provider* or of both sides if the study includes an actual interaction. For example, Bruneau and Saxe (2012) had Israelis and Palestinians converse with each other. They found that the attitudes of Israelis toward Palestinians became less extreme when they engaged in perspective-taking during the interaction. However, the Palestinians’ attitude toward the Israelis became less extreme when they received psychological safety by being listened to. This difference was attributed to the power differences between the groups. The Israelis were considered the high-power group (who may not consider the perspectives of others). In contrast, the Palestinians were considered the low-power group (who may not receive psychological safety from others). Thus, different groups have different emotional needs depending on their roles in a conflict (Shnabel et al., 2009), although research has not yet fully unpacked this issue.

Other studies have manipulated perspective-taking toward the target without an interaction. For example, Muradova and Arceneaux (2021) manipulated perspective-taking toward a person whose attitude was the opposite of the participants’ own attitude. The findings indicated that imagining the feelings and thoughts of the target facilitated more reflection and empathic feelings of concern, leading participants to shift their attitudes toward that individual. However, instructing people to give psychological safety by taking another person’s perspective may not necessarily increase accurate interpersonal understanding. Eyal et al. (2018) found that participants *perceived* that engaging in perspective-taking would increase their interpersonal accuracy. However, they found that in some conditions, perspective-taking *decreased* interpersonal accuracy in various tasks, including consumer attitudes, while increasing confidence in judgment. Their study highlights the importance of learning about another person’s perspective through a conversation rather than simply relying on preexisting knowledge or stereotypes. They found that getting another person’s perspective through conversation increased interpersonal accuracy, whereas utilizing existing knowledge without a conversation did not. Although engaging in perspective-taking can provide psychological safety to another person *via* increased sense of mutual understanding, it appears that perspective-taking that is responsive to an actual interaction partner is more likely to achieve understanding.

Perspective-taking can also be considered a self-persuasion technique. Self-persuasion occurs when individuals introspect on a topic and change their attitudes without input from external sources (Petty et al., 2003; Maio and Thomas, 2007). Most studies have found that perspective-taking promotes attitude change typically by prompting people to become more positive (or less negative) toward a person (or the person’s group) whose perspective has been taken (Tuller et al., 2015). However, Catapano et al. (2019) found that perspective-taking can backfire when disagreement is involved. Specifically, they found that individuals were less open to opposing views and less willing to change their attitudes when they had to put themselves in the shoes of a person who held an attitude that conflicted with theirs. Moreover, the backfiring effect of perspective-taking was amplified when individuals perceived the target person’s attitude as the opposite of their own but lessened when they took the perspective of someone who had similar values.

All the studies described above used instructed perspective-taking. Although adopting another’s perspective is one path to understanding them and providing them with psychological safety, this may not be a natural strategy people engage in, especially when others are dissimilar to the self. In studies with instructed perspective-taking, such as in Catapano et al. (2019), participants who put themselves in another person’s shoes by generating arguments opposite to their own values felt cognitive dissonance, which hindered their attitude change. Thus, people may not naturally take the perspective of someone too dissimilar from them. Furthermore, the richness of the input is likely to affect the impact of perspective-taking on attitude change. Specifically, perspective-taking might be easier with more complex and interactive stimuli such as through conversation or using virtual reality, because people can ask questions to “get” the perspective of others and thus understand them more (Herrera, 2020). On the other hand, minimal static stimuli may not allow for the rich perspective-taking that would promote attitude change more strongly.

## Interpersonal dynamics

Most of the studies discussed so far have focused on the effects of giving or receiving psychological safety on one side of an interaction. They often implement experimental paradigms to standardize the other half of the interaction to be consistent across conditions. This is a considerable strength from an experimental design standpoint, because it allows for much stronger statements about cause-and-effect relationships. However, the tradeoff is that this standardization limits the natural dynamics that can emerge in real-life interactions. Although not all of these dynamics have been studied in the context of attitudes and attitude change, the findings are strongly suggestive of the need to understand these dynamics if researchers want to understand how interactions affect people’s attitudes.

One key dynamic of real social interactions is reliance on reciprocity. There are strong social norms toward reciprocity in general (Gouldner, 1960). For example, when people are listened to well in dyadic conversations, they reciprocate by listening back to their conversation partner (Kluger et al., 2021). When the behavior that is reciprocated promotes psychological safety (e.g., asking questions to achieve a deeper understanding), the outcomes are very different from when the behavior that is reciprocated increases perceptions of threat (e.g., stating that one’s partner is “wrong”).

### Reciprocity

Reciprocity plays a significant role in interpersonal persuasion. Cialdini et al. (1992) found that when individuals acknowledge that their partner persuaded them, they end up being more likely to persuade their partner. Cialdini et al. (1992) argued that this could be at least partially attributed to the fact that the partner was reciprocating openness to influence. However, if initial resistance is acknowledged, partners reciprocate with resistance of their own.

One key aspect of reciprocity during conversations is question-asking. Suppose Kim asks Annie a question that shows interest in what Annie has said. In this case, Annie is likely to reciprocate by listening well to Kim and asking her good questions in return because question-asking expresses interest in others (Cojuharenco and Karelaia, 2020). This process can increase openness to attitude change. Chen et al. (2010) found that when individuals engaged in an online conversation that involved a disagreement, asking an elaboration question increased the favorability of people they converse with toward them and their willingness to engage in future conversations with them. The people they converse with also reciprocated by acting more receptive. Relatedly, when individuals used receptive language, they received less aggressive responses and prevented attitudinal conflicts from escalating (Yeomans et al., 2020). Yeomans et al. (2020) also found that the messages of communicators who exhibit receptiveness (i.e., provide psychological safety) were perceived as more persuasive. Finally, in speed-dating, individuals who asked their partners more questions were perceived with a more favorable attitude, and their interest was reciprocated by getting invited on more dates (Huang et al., 2017).

### Identity threat

When members of different ethnic groups interact, the self-identity of the socially disadvantaged group is often threatened because of concerns of being wrongly judged in a stereotyped manner (Shelton et al., 2006). When disadvantaged group members are concerned about being the target of prejudice from their interaction partners, they lose psychological safety, resulting in negative experiences (Shelton et al., 2005a). As a result of this threat, members of disadvantaged groups who interact with outgroup members who possess prejudiced attitudes experience impaired cognitive functioning (Murphy et al., 2012). Interestingly, societally advantaged group members often have concerns about being perceived as prejudiced, and as a result, their efforts to control the potential expression of prejudice can lead them to also feel cognitively and emotionally exhausted from the interaction (Richeson and Shelton, 2007). Furthermore, conditions for experiencing threats that might undermine safety appear to differ, at least for Black and White interactants in an American context (Trawalter and Richeson, 2008). The implications of these dynamics for an attitude change in interracial interactions have not been examined. However, the cognitive cost of these interactions could have a number of implications. For example, depleted individuals may not have resources to provide psychological safety to their partners, may be less able to resist persuasive messages (Wheeler et al., 2007; Burkley, 2008; Itzchakov et al., 2018b), and less able to generate messages of their own.

Thus, overall, understanding psychological safety in interpersonal interactions must consider the dynamics of interactions. Reciprocity norms and prejudice concerns that unfold over the course of an interaction constitute a subset of factors that can alter the trajectory of an interaction. Understanding these dynamics is needed to better grasp the implications for people’s attitudes in interpersonal contexts.

## General discussion

In this study, we reviewed attitude change from an interpersonal perspective and focused on the role of psychological safety in interactions. We noted that interpersonal interactions could pose a variety of psychological threats, which can lead people to behave inauthentically or defensively to deal with the threats. We then described how receiving psychological safety can reduce the threats and discussed implications for attitudes and attitude change. We reviewed the evidence on attitude change when individuals provide psychological safety. Finally, we reviewed the research on interpersonal dynamics such as reciprocity that can affect attitude change.

This review makes four contributions to the literature on attitude. First, it highlighted the importance of interpersonal processes and dynamics in attitude change that have been relatively neglected in traditional research on attitudes. Second, it highlighted ways in which attitudes can be changed in the absence of persuasive attempts, such as merely by adopting another person’s perspective or having another person provide psychological safety. Third, it posited psychological safety as a key unifying factor in many forms of influence in interpersonal contexts and highlights the crucial role psychological safety can play in promoting attitude change in interpersonal dynamics. Finally, it proposed several key consequences of psychological safety, including reduction in defensive processes and increase in self-reflection, intellectual humility, and openness, which may enable psychological safety to exert its effects on attitudes in interpersonal contexts.

Although we reviewed a wide range of studies and unified them under the umbrella of psychological safety, we believe that psychological safety as a construct has largely been overlooked in research on interpersonal persuasion. Seminal theories have suggested that when individuals feel psychologically safe, they become more open-minded, leading to possible attitudinal change (Rogers, 1980; Brehm and Brehm, 1981; Miller, 1983). In contrast, when psychological safety is lacking (especially if there is a threat), individuals tend to become more defensive, which reduces the likelihood that attitudes will change (Heller et al., 1973; Brehm and Brehm, 1981). A great deal of research has focused on psychological threats, but we hope this review will stimulate research to better understand psychological safety, particularly because it appears to attenuate or, at times, reverse many of the previously documented effects of threat.

Several topics were intentionally not reviewed in this study. These include compliance and conformity (Cialdini and Goldstein, 2004), behavioral mimicry (Mazar et al., 2022), distraction during persuasion (Petty et al., 1976), attitudes toward interaction partners or the groups they represent (Paluck et al., 2019), narrative persuasion (Green and Brock, 2002), and word-of-mouth persuasion (Herr et al., 1991). Although potentially relevant to interpersonal communication and attitude change, these topics were excluded because they do not generally involve receiving or providing psychological safety.

We believe that our study contributes to the literature on attitude and persuasion by positing psychological safety as a core organizational concept to understand how a range of interpersonal contexts may influence people’s attitudes. This review helps integrate otherwise disparate effects and processes that influence attitudes in interpersonal contexts. Importantly, it also sheds light on important unanswered questions that could guide future research.

### Unanswered questions and future directions

The vast majority of studies reviewed here focused on one side of interpersonal interaction (e.g., the individual who receives psychological safety or the individual who provides it). As noted above, paradigms that attempt to systematize one side of an interaction have huge benefits for causal inferences about the effects of psychological safety. These studies are also well-explained by existing theories of persuasion, such as the Elaboration Likelihood Model (Petty and Cacioppo, 1986), which could account for many of the results reviewed by positing that one key role that psychological safety can play is by leading to an increase in the extent of (unbiased) elaboration. Although some studies have examined the reciprocity of psychological safety-promoting behaviors (Lakey et al., 2010), examining attitude change in all the interactants is largely unexplored, particularly through the lens of psychological safety. For example, when a speaker receives psychological safety from a listener and consequently becomes less prejudiced or less extreme, do similar effects occur for the listener?

Given that social support constructs are usually reciprocated within the dyad (e.g., Malloy et al., 2021), listeners who observe speakers becoming more open-minded may also challenge their preexisting attitudes. Such effects may depend on the initial (in)congruence between the dyad members’ initial attitudes. Alternatively, because providing psychological safety is an effortful process that directs attention away from the self, providers of safety may not change their attitude at all because they do not have the cognitive and emotional capacities to introspect about it. If listeners do change their attitude, it might be because depletion or distraction opened them up to persuasion (e.g., Wheeler et al., 2007) but *via* different, lower elaboration processes than the changes facilitated by psychological safety.

In addition, although most of the research studies reviewed in this study deal with psychological safety in interpersonal interactions, threats to belongingness needs are also relevant. They have not been studied extensively in the context of attitudes and attitude change. As reviewed above, most attitude research studies related to interpersonal threats (e.g., belonging or one’s attitudes) have examined potential threats in anticipated interactions. Less is known about how threats in ongoing interactions (e.g., non-verbal cues of rejection or disapproval, verbal expressions of disagreement with important attitudes) can influence psychological and interpersonal processes and, hence, attitudes. It seems likely that just as safety can decrease defensive responding, such threats may also increase it. However, if there is a threat to inclusion or belongingness, are there ways in which people’s attempts to restore belongingness might differ from what would be predicted from the mere absence of safety? Almost certainly, the effects of such threats would depend on a host of other factors such as sense of psychological safety prior to the threat-inducing event and congeniality of the interactants’ attitudes. For example, when preexisting psychological safety exists in an interaction, a potential threat might be challenged or explicitly acknowledged (“I can tell from your facial expression that you disagree, why is that?”), whereas the same potential threat could lead to further disengagement and self-censorship in the absence of initial safety.

The effects of attitude change on interaction dynamics themselves have also received scant attention in the literature on attitudes. Prislin’s studies on influence in a group is a notable exception. For example, when speakers find that the attitudes of a group have shifted from initially opposed to being more congruent with their own views, the speakers’ confidence and comfort in the group increases (Prislin et al., 2011). Other studies have examined shifts in group consensus and found a host of effects on variables such as group identification and openness to dissent (Prislin et al., 2018). However, a small number of studies have examined how one person’s attitude change in a dyadic interaction affects aspects of the specific interaction and the relationship. As with group interactions, the effects of such shifts are likely to depend on the extent to which the new attitude is congruent with the interaction partners.

An additional open question is related to the stability of attitude change. The studies we reviewed primarily examined a single time point. Although this is a common practice in social psychological research, it is unclear whether and how long interpersonal attitude change can hold. Kalla and Broockman (2020) suggested that direct, non-judgmental interpersonal influence can be persistent. They obtained a lasting albeit small effect size of *d* = 0.08. Although this effect was small, it may not be atypical of “real world” (i.e., non-laboratory) attitude change, e.g., it is larger than the average effect of political campaign ads (Coppock et al., 2020). An exciting avenue to consider would be to compare this form of influence with impersonal persuasion. Because psychological safety leads to greater reflection on people’s attitudes, the increased thought might also increase attitude strength, as is characteristic of high elaboration conditions (Petty and Cacioppo, 1986).

Although intergroup relationships were beyond the scope of this review, it would be interesting to test psychological safety in these contexts. Numerous studies examined aspects of psychological safety in intergroup interactions to understand the experiences of interactants and identify conditions that promote a sense of safety in intergroup interactions (e.g., Milless et al., 2022). However, less research have examined whether and how these conditions extend to social influence. Although we would generally predict that psychological safety would reduce defensive responses and increase self-reflection, open-mindedness, and intellectual humility, we recognize that obstacles to achieving psychological safety are likely to be greater in intergroup than in same-group interactions. These barriers may vary depending on the specific historical or cultural context of the groups’ experiences in a given society and whether the topic is related to that context (e.g., a conversation about the merits of affirmative action policies). Furthermore, majority and minority group members may experience threats in different situations (Trawalter and Richeson, 2008) and may reciprocate each other’s experiences of threat and anxiety (West et al., 2009), which again underscores the need to understand interpersonal dynamics.

One key barrier to examining interpersonal dynamics is the methodological tools to do so. To adequately examine these dynamics, a host of variables should be considered, including overt behavior (e.g., question asking), momentary attitudes, and psychological experiences such as perceptions related to safety, threat, and receptivity (for a discussion of some possibilities with respect to receptivity, refer to Minson and Chen, 2021). One major challenge to collecting real-time data, and subjective perceptions in particular, is conducting such studies without disrupting the flow of the conversation. Beyond the dynamics of the dyadic interaction, researchers also need to take broader dynamics and networks that may be relevant to understanding people’s attitudes and their interaction goals and expectations into account (Smith and Semin, 2004). For example, a discussion among members of opposing political parties, even if marked by psychological safety, is difficult to understand without understanding the political identities and dynamics that shaped their initial attitudes and will do so again once the interaction has ended.

This review suggests that both psychological safety, which promotes open-mindedness, and self-threat, which reduces open-mindedness, can create attitude change under some (although not the same) conditions. Hence, future research should examine the moderators that account for the attitudinal effects of these two different intrapersonal processes. The properties of people’s attitudes might be one potential set of moderators. For example, the attitudes of speakers who had one-sided initial attitudes were less likely to change than those who initially had two-sided attitudes in response to high-quality listening (Itzchakov et al., 2017; Study 4). On the other hand, paradoxical thinking, which operates by increasing self-threat for its recipients, has mostly been studied in the case of strong attitudes such as political attitudes. The findings suggest that the intervention is more effective for people with extreme (i.e. one-sided) attitudes (Hameiri et al., 2018). Thus, initial evidence suggests that at least some interpersonal influence techniques depend on the properties of people’s initial attitudes. However, very little research on this topic has been conducted, and only a small subset of potentially relevant attitudinal properties has been investigated.

Another intriguing issue is how interpersonal context affects the creation and reception of a persuasive message. For example, how does a sense of psychological safety change the messages produced? Given that psychological safety seems to increase open-mindedness and intellectual humility, are people less likely to adopt forceful language in their messages and more likely to use hedges? How might people’s interaction goals (e.g., to persuade vs. to form a relationship) affect message generation, and how might differences in messages affect perceptions of psychological safety? In addition, do people’s lay theories of influence in interactions map onto reality? Certainly, anecdotal evidence suggests that people often adopt approaches that threaten psychological safety, such as forceful, persuasion-oriented approaches to topics they care greatly about (e.g., merits of vaccination). Such approaches lead to conflict and attitude entrenchment rather than intended change. Does shifting people’s goals or mindsets for interactions change the interpersonal dynamics as well as the outcomes for people’s attitudes?

Finally almost all research on interpersonal influence has been done in western cultures, where independent self-construals (Markus and Kitayama, 2010) and loose social norms predominate (Gelfand et al., 2006), and in which individual attitudes are of high importance. Little is known about how well the principles observed in western cultures would extend to more collectivistic cultures or cultures with stricter social norms. Examining a wider range of cultural contexts is critical for understanding interpersonal social influence, because culture shapes the expectations, norms, and scripts of interactions (Kirkman et al., 2016). People from different cultures may respond differently to the same behaviors in an interaction because of the culture-specific meaning of those behaviors. Culture may also shape the meaning and importance of people’s individual attitudes relative to information from the immediate social context, thus shifting the potential constructs of interest when exploring the effects of influence in interpersonal contexts (Riemer et al., 2014). Future research should begin to investigate influence in interpersonal contexts across cultures by examining whether similar effects are observed in different cultural contexts and, if so, whether similar psychological mechanisms are responsible for these effects.

### Limitations of current approaches

Many of the studies reviewed here did not include actual or virtual interactions. Even though some classic studies involved direct interactions among participants (e.g., Janis and King, 1954), such paradigms are not often used in contemporary attitude research. Most studies analyzing interpersonal processes in persuasion have used imagined or anticipated interactions. This is understandable from a practical perspective. Face-to-face interactions are hard to conduct and require greater logistical and financial resources. These difficulties often run counter to contemporary demands for greater statistical power (for a discussion, refer to Finkel et al., 2015).

Nevertheless, the major problem is that virtual and imagined interactions lack ecological validity and limit or eliminate interpersonal dynamics. Critically, people’s expectations, attitudes, and predictions about how they will behave do not always align with their actual behavior (Webb and Sheeran, 2006; Kawakami et al., 2009), making it difficult to generalize from imagined interactions to real ones. One study that explicitly examined differences between real interactions and video presentation of the same information found meaningful differences (Norton et al., 2012). However, despite the challenges of conducting research involving real interactions, we believe that the potential importance of the knowledge to be gained – both in terms of our fundamental understanding of psychological and interpersonal processes and society more broadly – makes engaging in this research a worthwhile endeavor.

## Conclusion

This review sheds light on psychological safety as an important ingredient of interpersonal attitude change. Receiving or providing psychological safety can lead to a variety of psychological processes that can influence attitude change, which often leads to moderation of people’s opinions. The findings suggest that psychological safety can affect people’s attitudes even in the absence of an attempt to persuade. Although there is a great deal of interesting research studies examining influence in interpersonal contexts, the literature is incomplete and is limited in a number of important ways as described above. We hope that this review will stimulate more research studies on this important topic.

## Author contributions

GI and KD planned the review outline and wrote the manuscript together. Both authors contributed to the article and approved the submitted version.

## Footnotes

1 This specific study did not examine attitude correctness, but based on previously cited studies, correctness is the most likely form of certainty that would be affected in these conditions.

## References

[B1] Argyris C. Schon D. A. (1978). *Organizational Learning.* Reading, MA: Addison-Wesley.

[B2] Bar-Tal D. Hameiri B. Halperin E. (2021). “Paradoxical thinking as a paradigm of attitude change in the context of intractable conflict,” in *Advances in Experimental Social Psychology*, Vol. 63 ed. Gawronski B. (Amsterdam: Elsevier), 129–187. 10.1016/bs.aesp.2020.11.003

[B3] Baumeister R. F. Leary M. R. (1995). The need to belong: desire for interpersonal attachments as a fundamental human motivation. *Psychol. Bull.* 117 497–529. 10.1037/0033-2909.117.3.497 7777651

[B4] Berkovich I. Eyal O. (2018). The effects of principals’ communication practices on teachers’ emotional distress. *Educ. Manage. Adm. Leadersh.* 46 642–658. 10.1177/1741143217694894

[B5] Brehm S. S. Brehm J. W. (1981). *Psychological Reactance: A Theory of Freedom and Control.* New York, NY: Academic Press.

[B6] Briñol P. Petty R. E. Gallardo I. DeMarree K. G. (2007). The effect of self-affirmation in nonthreatening persuasion domains: timing affects the process. *Pers. Soc. Psychol. Bull.* 33 1533–1546. 10.1177/0146167207306282 17933742

[B7] Bruneau E. G. Saxe R. (2012). The power of being heard: the benefits of ‘perspective-giving’ in the context of intergroup conflict. *J. Exp. Soc. Psychol.* 48 855–866. 10.1016/j.jesp.2012.02.017

[B8] Burkley E. (2008). The role of self-control in resistance to persuasion. *Pers. Soc. Psychol. Bull.* 34 419–431. 10.1177/0146167207310458 18272808

[B9] Byrne D. (1961). Interpersonal attraction and attitude similarity. *J. Abnorm. Soc. Psychol.* 62 713–715. 10.1037/h0044721 13875334

[B10] Carmeli A. Reiter-Palmon R. Ziv E. (2010). Inclusive leadership and employee involvement in creative tasks in the workplace: the mediating role of psychological safety. *Creat. Res. J.* 22 250–260. 10.1080/10400419.2010.504654

[B11] Castro D. R. Kluger A. N. Itzchakov G. (2016). Does avoidance-attachment style attenuate the benefits of being listened to? *Eur. J. Soc. Psychol.* 46 762–775. 10.1002/ejsp.2185

[B12] Catapano R. Tormala Z. L. Rucker D. D. (2019). Perspective taking and self-persuasion: why “Putting Yourself in Their Shoes” reduces openness to attitude change. *Psychol. Sci.* 30 424–435. 10.1177/0956797618822697 30694721

[B13] Chaiken S. Liberman A. Eagly A. H. (1989). “Heuristic and systematic information processing within and beyond the persuasion context,” in *Unintended Thought*, eds Uleman J. S. Bargh J. A. (New York, NY: Guilford Press), 212–252.

[B14] Cheatham L. Tormala Z. L. (2015). Attitude certainty and attitudinal advocacy: the unique roles of clarity and correctness. *Pers. Soc. Psychol. Bull.* 41 1537–1550. 10.1177/0146167215601406 26338853

[B15] Chen F. S. Minson J. A. Tormala Z. L. (2010). Tell me more: the effects of expressed interest on receptiveness during dialog. *J. Exp. Soc. Psychol.* 46 850–853. 10.1016/j.jesp.2010.04.012

[B16] Cho Y. (2022). Team diversity, perspective taking, and employee creativity: the importance of psychological safety. *Soc. Behav. Pers.* 50:e11042. 10.2224/sbp.11042

[B17] Cialdini R. B. Goldstein N. J. (2004). Social influence: compliance and conformity. *Annu. Rev. Psychol.* 55 591–621. 10.1146/annurev.psych.55.090902.142015 14744228

[B18] Cialdini R. B. Green B. L. Rusch A. J. (1992). When tactical pronouncements of change become real change: the case of reciprocal persuasion. *J. Pers. Soc. Psychol.* 63 30–40. 10.1037/0022-3514.63.1.30

[B19] Cialdini R. B. Levy A. Herman C. P. Evenbeck S. (1973). Attitudinal politics: the strategy of moderation. *J. Pers. Soc. Psychol.* 25 100–108. 10.1037/h0034265

[B20] Cialdini R. B. Levy A. Herman C. P. Kozlowski L. T. Petty R. E. (1976). Elastic shifts of opinion: determinants of direction and durability. *J. Pers. Soc. Psychol.* 34 663–672. 10.1037/0022-3514.34.4.663

[B21] Cojuharenco I. Karelaia N. (2020). When leaders ask questions: can humility premiums buffer the effects of competence penalties? *Organ. Behav. Hum. Decis. Process.* 156 113–134. 10.1016/j.obhdp.2019.12.001

[B22] Coppock A. Hill S. J. Vavreck L. (2020). The small effects of political advertising are small regardless of context, message, sender, or receiver: evidence from 59 real-time randomized experiments. *Sci. Adv.* 6:eabc4046. 10.1126/sciadv.abc4046 32917601 PMC7467695

[B23] Critcher C. R. Dunning D. (2015). Self-affirmations provide a broader perspective on self-threat. *Pers. Soc. Psychol. Bull.* 41 3–18. 10.1177/0146167214554956 25319717

[B24] Edmondson A. (1999). Psychological safety and learning behavior in work teams. *Adm. Sci. Q.* 44 350–383. 10.2307/2666999

[B25] Edmondson A. C. Lei Z. (2014). Psychological safety: the history, renaissance, and future of an interpersonal construct. *Annu. Rev. Organ. Psychol. Organ. Behav.* 1 23–43. 10.1146/annurev-orgpsych-031413-091305

[B26] Epton T. Harris P. R. Kane R. van Koningsbruggen G. M. Sheeran P. (2015). The impact of self-affirmation on health-behavior change: a meta-analysis. *Health Psychol.* 34 187–196. 10.1037/hea0000116 25133846

[B27] Eyal T. Steffel M. Epley N. (2018). Perspective mistaking: accurately understanding the mind of another requires getting perspective, not taking perspective. *J. Pers. Soc. Psychol.* 114 547–571. 10.1037/pspa0000115 29620401

[B28] Finkel E. J. Eastwick P. W. Reis H. T. (2015). Best research practices in psychology: illustrating epistemological and pragmatic considerations with the case of relationship science. *J. Pers. Soc. Psychol.* 108 275–297. 10.1037/pspi0000007 25603376

[B29] Frimer J. A. Skitka L. J. (2020). Are politically diverse Thanksgiving dinners shorter than politically uniform ones? *PLoS One* 15:e0239988. 10.1371/journal.pone.0239988 33108382 PMC7591091

[B30] Gelfand M. J. Nishii L. H. Raver J. L. (2006). On the nature and importance of cultural tightness-looseness. *J. Appl. Psychol.* 91 1225–1244. 10.1037/0021-9010.91.6.1225 17100480

[B31] Gordon A. M. Chen S. (2016). Do you get where I’m coming from?: Perceived understanding buffers against the negative impact of conflict on relationship satisfaction. *J. Pers. Soc. Psychol.* 110 239–260. 10.1037/pspi0000039 26523997

[B32] Gouldner A. W. (1960). The norm of reciprocity: a preliminary statement. *Am. Sociol. Rev.* 25 161–178. 10.2307/2092623

[B33] Green M. C. Brock T. C. (2002). “In the mind’s eye: transportation-imagery model of narrative persuasion,” in *Narrative Impact: Social and Cognitive Foundations*, eds Green M. C. Strange J. J. Brock T. C. (Mahwah, NJ: Lawrence Erlbaum Associates Publishers), 315–341. 10.4324/9781410606648

[B34] Hameiri B. Nabet E. Bar-Tal D. Halperin E. (2018). Paradoxical thinking as a conflict-resolution intervention: comparison to alternative interventions and examination of psychological mechanisms. *Pers. Soc. Psychol. Bull.* 44 122–139. 10.1177/0146167217736048 29052459

[B35] Hameiri B. Porat R. Bar-Tal D. Bieler A. Halperin E. (2014). Paradoxical thinking as a new avenue of intervention to promote peace. *Proc. Natl. Acad. Sci. U.S.A.* 111 10996–11001. 10.1073/pnas.1407055111 25024185 PMC4121792

[B36] Han D. Duhachek A. Rucker D. D. (2015). Distinct threats, common remedies: how consumers cope with psychological threat. *J. Consum. Psychol.* 25 531–545. 10.1016/j.jcps.2015.02.001

[B37] Heller J. F. Pallak M. S. Picek J. M. (1973). The interactive effects of intent and threat on boomerang attitude change. *J. Pers. Soc. Psychol.* 26 273–279. 10.1037/h0034461

[B38] Herr P. M. Kardes F. R. Kim J. (1991). Effects of word-of-mouth and product-attribute information on persuasion: an accessibility-diagnosticity perspective. *J. Consum. Res.* 17 454–462. 10.1086/208570

[B39] Herrera F. (2020). “Virtual embodiment and embodied cognition: effect of virtual reality perspective taking tasks on empathy and prejudice,” in *A Multidisciplinary Approach to Embodiment*, ed. Dess N. K. (New York, NY: Routledge), 127–132. 10.4324/9780429352379-26

[B40] Huang K. Yeomans M. Brooks A. W. Minson J. Gino F. (2017). It doesn’t hurt to ask: question-asking increases liking. *J. Pers. Soc. Psychol.* 113 430. 10.1037/pspi0000097 28447835

[B41] Hussein M. A. Tormala Z. L. (2021). Undermining your case to enhance your impact: a framework for understanding the effects of acts of receptiveness in persuasion. *Pers. Soc. Psychol. Rev.* 25 229–250. 10.1177/10888683211001269 33813983

[B42] Itzchakov G. Castro D. R. Kluger A. N. (2016). If you want people to listen to you, tell a story. *Int. J. Listen.* 30 120–133. 10.1080/10904018.2015.1037445

[B43] Itzchakov G. DeMarree K. G. Kluger A. N. Turjeman-Levi Y. (2018a). The listener sets the tone: high-quality listening increases attitude clarity and behavior-intention consequences. *Pers. Soc. Psychol. Bull.* 44 762–778. 10.1177/0146167217747874 29347879

[B44] Itzchakov G. Uziel L. Wood W. (2018b). When attitudes and habits don’t correspond: self-control depletion increases persuasion but not behavior. *J. Exp. Soc. Psychol.* 75 1–10. 10.1016/j.jesp.2017.10.011

[B45] Itzchakov G. Kluger A. N. (2017). Can holding a stick improve listening at work? The effect of listening circles on employees’ emotions and cognitions. *Eur. J. Work Organ. Psychol.* 26 663–676. 10.1080/1359432X.2017.1351429

[B46] Itzchakov G. Kluger A. N. Castro D. R. (2017). I am aware of my inconsistencies but can tolerate them: the effect of high quality listening on speakers’ attitude ambivalence. *Pers. Soc. Psychol. Bull.* 43 105–120. 10.1177/0146167216675339 27856728

[B47] Itzchakov G. Reis H. T. (2021). Perceived responsiveness increases tolerance of attitude ambivalence and enhances intentions to behave in an open-minded manner. *Pers. Soc. Psychol. Bull.* 47 468–485. 10.1177/0146167220929218 32552420

[B48] Itzchakov G. Weinstein N. Legate N. Amar M. (2020). Can high quality listening predict lower speakers’ prejudiced attitudes? *J. Exp. Soc. Psychol.* 91:104022. 10.1016/j.jesp.2020.104022 32834106 PMC7409873

[B49] Itzchakov G. Reis H. T. Weinstein N. (2022a). How to foster perceived partner responsiveness: high-quality listening is key. *Soc. Pers. Psychol. Compass* 16:e12648. 10.1111/spc3.12648

[B50] Itzchakov G. Weinstein N. Saluk D. Amar M. (2022b). Connection heals wounds: feeling listened to reduces speakers’ loneliness following a social rejection disclosure. *Pers. Soc. Psychol. Bull.* 01461672221100369. 10.1177/01461672221100369 [Epub ahead of print]. 35726696 PMC10320710

[B51] Janis I. L. (1972). *Victims of Groupthink.* Boston, MA: Houghton-Mifflin.

[B52] Janis I. L. King B. T. (1954). The influence of role playing on opinion change. *J. Abnorm. Soc. Psychol.* 49 211–218. 10.1037/h0056957 13151774

[B53] Jones E. E. Rhodewalt F. Berglas S. Skelton J. A. (1981). Effects of strategic self-presentation on subsequent self-esteem. *J. Pers. Soc. Psychol.* 41 407–421. 10.1037/0022-3514.41.3.407

[B54] Kalla J. L. Broockman D. E. (2020). Reducing exclusionary attitudes through interpersonal conversation: evidence from three field experiments. *Am. Polit. Sci. Rev.* 114 410–425. 10.1017/S0003055419000923

[B55] Kark R. Carmeli A. (2009). Alive and creating: the mediating role of vitality and aliveness in the relationship between psychological safety and creative work involvement. *J. Organ. Behav.* 30 785–804. 10.1002/job.571

[B56] Kawakami K. Dunn E. Karmali F. Dovidio J. F. (2009). Mispredicting affective and behavioral responses to racism. *Science* 323 276–278. 10.1126/science.1164951 19131633

[B57] Kirkman B. L. Shapiro D. L. Lu S. McGurrin D. P. (2016). Culture and teams. *Curr. Opin. Psychol.* 8 137–142. 10.1016/j.copsyc.2015.12.001 29506789

[B58] Kluger A. N. Itzchakov G. (2022). The power of listening at work. *Annu. Rev. Organ. Psychol. Organ. Behav.* 9 121–146. 10.1146/annurev-orgpsych-012420-091013

[B59] Kluger A. N. Malloy T. E. Pery S. Itzchakov G. Castro D. R. Lipetz L. (2021). Dyadic listening in teams: social relations model. *Appl. Psychol.* 70 1045–1099. 10.1111/apps.12263

[B60] Kunda Z. (1990). The case for motivated reasoning. *Psychol. Bull.* 108 480–498. 10.1037/0033-2909.108.3.480 2270237

[B61] Lakey B. Orehek E. Hain K. L. VanVleet M. (2010). Enacted support’s links to negative affect and perceived support are more consistent with theory when social influences are isolated from trait influences. *Pers. Soc. Psychol. Bull.* 36 132–142. 10.1177/0146167209349375 19875827

[B62] Leary M. Gabriel S. (2022). The relentless pursuit of acceptance and belonging. *Adv. Motiv. Sci.* 9 135–178. 10.1016/bs.adms.2021.12.001

[B63] Leary M. R. Diebels K. J. Davisson E. K. Jongman-Sereno K. P. Isherwood J. C. Raimi K. T. (2017). Cognitive and interpersonal features of intellectual humility. *Pers. Soc. Psychol. Bull.* 43 793–813. 10.1177/0146167217697695 28903672

[B64] Lepper M. R. Woolverton M. (2002). “The wisdom of practice: lessons learned from the study of highly effective tutors,” in *Improving Academic Achievement*, ed. Aronson J. (Amsterdam: Elsevier), 135–158. 10.1016/B978-012064455-1/50010-5

[B65] Li L. Zhu S. Tse N. Tse S. Wong P. (2016). Effectiveness of motivational interviewing to reduce illicit drug use in adolescents: a systematic review and meta-analysis. *Addiction* 111 795–805. 10.1111/add.13285 26687544

[B66] Luttrell A. Sawicki V. (2020). Attitude strength: distinguishing predictors versus defining features. *Soc. Pers. Psychol. Compass* 14:e12555. 10.1111/spc3.12555

[B67] Maio G. R. Haddock G. Verplanken B. (2018). *The Psychology of Attitudes and Attitude Change.* Thousand Oaks, CA: Sage Publications Limited.

[B68] Maio G. R. Thomas G. (2007). The epistemic-teleologic model of deliberate self-persuasion. *Pers. Soc. Psychol. Rev.* 11 46–67. 10.1177/1088868306294589 18453455

[B69] Malloy T. E. Kluger A. N. Martin J. Pery S. (2021). Women listening to women at zero-acquaintance: interpersonal befriending at the individual and dyadic levels. *Int. J. Listen.* 1–15. 10.1080/10904018.2021.1884080

[B70] Markus H. R. Kitayama S. (2010). Cultures and selves: a cycle of mutual constitution. *Perspect. Psychol. Sci.* 5 420–430. 10.1177/1745691610375557 26162188

[B71] Mazar A. Itzchakov G. Lieberman A. Wood W. (2022). The unintentional nonconformist: habits promote resistance to social influence. *Pers. Soc. Psychol. Bull.* 10.1177/01461672221086177 [Epub ahead of print]. 35485353

[B72] Miller W. R. (1983). Motivational interviewing with problem drinkers. *Behav. Cogn. Psychother.* 11 147–172. 10.1017/S0141347300006583

[B73] Miller W. R. Rose G. S. (2009). Toward a theory of motivational interviewing. *Am. Psychol.* 64 527–537. 10.1037/a0016830 19739882 PMC2759607

[B74] Miller W. R. Rose G. S. (2015). Motivational interviewing and decisional balance: contrasting responses to client ambivalence. *Behav. Cogn. Psychother.* 43 129–141. 10.1017/S1352465813000878 24229732

[B75] Milless K. L. Wout D. A. Murphy M. C. (2022). Diversity or representation? Sufficient factors for Black Americans’ identity safety during interracial interactions. *Cult. Divers. Ethnic Minor. Psychol.* 28 103–111. 10.1037/cdp0000492 34807671

[B76] Minson J. A. Chen F. S. (2021). Receptiveness to opposing views: conceptualization and integrative review. *Pers. Soc. Psychol. Rev.* 26 93–111. 10.1177/10888683211061037 34964408

[B77] Montoya R. M. Horton R. S. Kirchner J. (2008). Is actual similarity necessary for attraction? A meta-analysis of actual and perceived similarity. *J. Soc. Pers. Relat.* 25 889–922. 10.1177/0265407508096700

[B78] Muradova L. Arceneaux K. (2021). Reflective political reasoning: political disagreement and empathy. *Eur. J. Polit. Res.* 61, 740–761. 10.1111/1475-6765.12490

[B79] Murphy M. C. Richeson J. A. Shelton J. N. Rheinschmidt M. L. Bergsieker H. B. (2012). Cognitive costs of contemporary prejudice. *Group Process. Intergroup Relat.* 16 560–571. 10.1177/1368430212468170

[B80] Norton M. I. Dunn E. W. Carney D. R. Ariely D. (2012). The persuasive “power” of stigma? *Organ. Behav. Hum. Decis. Process.* 117 261–268. 10.1016/j.obhdp.2011.08.002

[B81] O’Donovan R. McAuliffe E. (2020). A systematic review exploring the content and outcomes of interventions to improve psychological safety, speaking up and voice behaviour. *BMC Health Serv. Res.* 20:101. 10.1186/s12913-020-4931-2 32041595 PMC7011517

[B82] Paluck E. L. Green S. A. Green D. P. (2019). The contact hypothesis re-evaluated. *Behav. Public Policy* 3 129–158. 10.1017/bpp.2018.25

[B83] Petrocelli J. V. Tormala Z. L. Rucker D. D. (2007). Unpacking attitude certainty: attitude clarity and attitude correctness. *J. Pers. Soc. Psychol.* 92 30–41. 10.1037/0022-3514.92.1.30 17201540

[B84] Petty R. E. Cacioppo J. T. (1986). “The elaboration likelihood model of persuasion,” in *Advances in Experimental Social Psychology*, Vol. 19, ed. Berkowitz L. (New York, NY: Academic Press), 123–205.

[B85] Petty R. E. Wells G. L. Brock T. C. (1976). Distraction can enhance or reduce yielding to propaganda: thought disruption versus effort justification. *J. Pers. Soc. Psychol.* 34 874–884. 10.1037/0022-3514.34.5.874

[B86] Petty R. E. Wheeler S. C. Tormala Z. L. (2003). “Persuasion and attitude change,” in *Handbook of Psychology: Personality and Social Psychology*, Vol. 5 eds Millon T. Lerner M. J. (Hoboken, NJ: John Wiley & Sons, Inc), 353–382. 10.1002/0471264385.wei0515

[B87] Pillaud V. Cavazza N. Butera F. (2013). The social value of being ambivalent: self-presentational concerns in the expression of attitudinal ambivalence. *Pers. Soc. Psychol. Bull.* 39 1139–1151. 10.1177/0146167213490806 23761925

[B88] Pillaud V. Cavazza N. Butera F. (2018). The social utility of ambivalence: being ambivalent on controversial issues is recognized as competence. *Front. Psychol.* 9:961. 10.3389/fpsyg.2018.00961 29988468 PMC6024988

[B89] Priester J. R. Petty R. E. (1996). The gradual threshold model of ambivalence: relating the positive and negative bases of attitudes to subjective ambivalence. *J. Pers. Soc. Psychol.* 71 431–449. 10.1037/0022-3514.71.3.431 8831157

[B90] Prislin R. Christensen P. N. (2002). Group conversion versus group expansion as modes of change in majority and minority positions: all losses hurt but only some gains gratify. *J. Pers. Soc. Psychol.* 83 1095–1102. 10.1037/0022-3514.83.5.1095 12416914

[B91] Prislin R. Davenport C. Xu Y. Moreno R. Honeycutt N. (2018). From marginal to mainstream and vice versa: leaders’ valuation of diversity while in the minority versus majority. *J. Soc. Issues* 74 112–128. 10.1111/josi.12259

[B92] Prislin R. Limbert W. M. Bauer E. (2000). From majority to minority and vice versa: the asymmetrical effects of losing and gaining majority position within a group. *J. Pers. Soc. Psychol.* 79 385–397. 10.1037/0022-3514.79.3.385 10981841

[B93] Prislin R. Sawicki V. Williams K. (2011). New majorities’ abuse of power: effects of perceived control and social support. *Group Process. Intergroup Relat.* 14 489–504. 10.1177/1368430210391310

[B94] Reis H. T. (2012). “Perceived partner responsiveness as an organizing theme for the study of relationships and well-being,” in *Interdisciplinary Research on Close Relationships: The Case for Integration*, eds Campbell L. Loving T. J. (Washington, DC: American Psychological Association), 27–52. 10.1037/13486-002

[B95] Reis H. T. Clark M. S. Holmes J. G. (2004). “Perceived partner responsiveness as an organizing construct in the study of intimacy and closeness,” in *Handbook of Closeness and Intimacy*, eds Mashek D. J. Aron A. P. (Mahwah, NJ: Lawrence Erlbaum Associates Publishers), 201–225.

[B96] Reis H. T. Gable S. L. (2003). “Toward a positive psychology of relationships,” in *Flourishing: Positive Psychology and the Life Well-Lived*, eds Keyes C. L. M. Haidt J. (Washington DC: American Psychological Association), 129–159. 10.1037/10594-006

[B97] Reis H. T. Lee K. Y. O’Keefe S. D. Clark M. S. (2018). Perceived partner responsiveness promotes intellectual humility. *J. Exp. Soc. Psychol.* 79 21–33. 10.1016/j.jesp.2018.05.006

[B98] Reis H. T. Lemay E. P. Finkenauer C. (2017). Toward understanding understanding: the importance of feeling understood in relationships. *Soc. Pers. Psychol. Compass* 11:e12308. 10.1111/spc3.12308

[B99] Rhodewalt F. Agustsdottir S. (1986). Effects of self-presentation on the phenomenal self. *J. Pers. Soc. Psychol.* 50 47–55. 10.1037/0022-3514.50.1.47

[B100] Richeson J. A. Shelton J. N. (2007). Negotiating interracial interactions: costs, consequences, and possibilities. *Curr. Dir. Psychol. Sci.* 16 316–320. 10.1111/j.1467-8721.2007.00528.x

[B101] Riemer H. Shavitt S. Koo M. Markus H. R. (2014). Preferences don’t have to be personal: expanding attitude theorizing with a cross-cultural perspective. *Psychol. Rev.* 121 619–648. 10.1037/a0037666 25347311

[B102] Rios K. DeMarree K. G. Statzer J. (2014). Attitude certainty and conflict style: divergent effects of correctness and clarity. *Pers. Soc. Psychol. Bull.* 40 819–830. 10.1177/0146167214528991 24727810

[B103] Rogers C. R. (1980). *A Way of Being.* Boston, MA: Houghton Mifflin.

[B104] Rosenberg B. D. Siegel J. T. (2021). Threatening uncertainty and psychological reactance: are freedom threats always noxious? *Curr. Psychol.* 1–10. 10.1007/s12144-021-01640-8 [Epub ahead of print].

[B105] Schlosser A. Shavitt S. (1999). Effects of an approaching group discussion on product responses. *J. Consum. Psychol.* 8 377–406. 10.1207/s15327663jcp0804_02

[B106] Schlosser A. E. Shavitt S. (2002). Anticipating discussion about a product: rehearsing what to say can affect your judgments. *J. Consum. Res.* 29 101–115. 10.1086/339924

[B107] Schwarz N. (2007). Attitude construction: evaluation in context. *Soc. Cogn.* 25 638–656. 10.1521/soco.2007.25.5.638

[B108] Shelton J. N. Richeson J. A. Salvatore J. Trawalter S. (2005b). Ironic effects of racial bias during interracial interactions. *Psychol. Sci.* 16 397–402. 10.1111/j.0956-7976.2005.01547.x 15869700

[B109] Shelton J. N. Richeson J. A. Salvatore J. (2005a). Expecting to be the target of prejudice: implications for interethnic interactions. *Pers. Soc. Psychol. Bull.* 31 1189–1202. 10.1177/0146167205274894 16055639

[B110] Shelton J. N. Richeson J. A. Vorauer J. D. (2006). Threatened identities and interethnic interactions. *Eur. Rev. Soc. Psychol.* 17 321–358. 10.1080/10463280601095240

[B111] Sherf E. N. Parke M. R. Isaakyan S. (2021). Distinguishing voice and silence at work: unique relationships with perceived impact, psychological safety, and burnout. *Acad. Manage. J.* 64 114–148. 10.5465/amj.2018.1428

[B112] Sherman D. A. Nelson L. D. Steele C. M. (2000). Do messages about health risks threaten the self? Increasing the acceptance of threatening health messages via self-affirmation. *Pers. Soc. Psychol. Bull.* 26 1046–1058. 10.1177/01461672002611003

[B113] Sherman D. K. Cohen G. L. (2002). Accepting threatening information: self–affirmation and the reduction of defensive biases. *Curr. Dir. Psychol. Sci.* 11 119–123. 10.1111/1467-8721.00182

[B114] Sherman D. K. Cohen G. L. (2006). “The psychology of self-defense: self-affirmation theory,” in *Advances in Experimental Social Psychology*, Vol. 38 ed. Zanna M. P. (Cambridge, MA: Academic Press), 183–242. 10.1016/S0065-2601(06)38004-5

[B115] Shnabel N. Nadler A. Ullrich J. Dovidio J. F. Carmi D. (2009). Promoting reconciliation through the satisfaction of the emotional needs of victimized and perpetrating group members: the needs-based model of reconciliation. *Pers. Soc. Psychol. Bull.* 35 1021–1030. 10.1177/0146167209336610 19498070

[B116] Smith E. R. Semin G. R. (2004). “Socially situated cognition: cognition in its social context,” in *Advances in Experimental Social Psychology*, Vol. 36 ed. Zanna M. P. (Amsterdam: Elsevier), 53–117. 10.1016/S0065-2601(04)36002-8

[B117] Steele C. M. (1988). “The psychology of self-affirmation: sustaining the integrity of the self,” in *Advances in Experimental Social Psychology*, Vol. 21 ed. Berkowitz L. (Cambridge, MA: Academic Press), 261–302. 10.1016/S0065-2601(08)60229-4

[B118] Stone J. Whitehead J. Schmader T. Focella E. (2011). Thanks for asking: Self-affirming questions reduce backlash when stigmatized targets confront prejudice. *J. Exp. Soc. Psychol.* 47 589–598. 10.1016/j.jesp.2010.12.016

[B119] Swann W. B. J. (1990). “To be adored or to be known: the interplay of self-enhancement and self-verification,” in *Handbook of Motivation and Cognition*, Vol. 2 eds Sorrentino R. Higgins E. T. (New York, NY: Guilford), 408–448.

[B120] Tetlock P. E. Skitka L. Boettger R. (1989). Social and cognitive strategies for coping with accountability: conformity, complexity, and bolstering. *J. Pers. Soc. Psychol.* 57 632–640. 10.1037/0022-3514.57.4.632 2795435

[B121] Trawalter S. Richeson J. A. (2008). Let’s talk about race, baby! When whites’ and blacks’ interracial contact experiences diverge. *J. Exp. Soc. Psychol.* 44 1214–1217. 10.1016/j.jesp.2008.03.013 19578470 PMC2493421

[B122] Tuller H. M. Bryan C. J. Heyman G. D. Christenfeld N. J. (2015). Seeing the other side: perspective taking and the moderation of extremity. *J. Exp. Soc. Psychol.* 59 18–23. 10.1016/j.jesp.2015.02.003

[B123] Tynan R. (2005). The effects of threat sensitivity and face giving on dyadic psychological safety and upward communication 1. *J. Appl. Soc. Psychol.* 35 223–247. 10.1111/j.1559-1816.2005.tb02119.x

[B124] Van Quaquebeke N. Felps W. (2018). Respectful inquiry: a motivational account of leading through asking questions and listening. *Acad. Manage. Rev.* 43 5–27. 10.5465/amr.2014.0537

[B125] Voelkel J. G. Ren D. Brandt M. J. (2021). Inclusion reduces political prejudice. *J. Exp. Soc. Psychol.* 95:104149. 10.1016/j.jesp.2021.104149

[B126] Waldron V. R. Kelley D. L. (2005). Forgiving communication as a response to relational transgressions. *J. Soc. Pers. Relat.* 22 723–742. 10.1177/0265407505056445

[B127] Webb T. L. Sheeran P. (2006). Does changing behavioral intentions engender behavior change? A meta-analysis of the experimental evidence. *Psychol. Bull.* 132 249–268. 10.1037/0033-2909.132.2.249 16536643

[B128] Weinstein N. Huo A. Itzchakov G. (2021). Parental listening when adolescents self-disclose: a preregistered experimental study. *J. Exp. Child Psychol.* 209:105178. 10.1016/j.jecp.2021.105178 34087604

[B129] Weinstein N. Itzchakov G. Legate N. (2022). The motivational value of listening during intimate and difficult conversations. *Soc. Pers. Psychol. Compass* 16:e12651. 10.1111/spc3.12651

[B130] West T. V. Shelton J. N. Trail T. E. (2009). Relational anxiety in interracial interactions. *Psychol. Sci.* 20 289–292. 10.1111/j.1467-9280.2009.02289.x 19207693

[B131] Wheeler S. C. Briñol P. Hermann A. D. (2007). Resistance to persuasion as self-regulation: ego-depletion and its effects on attitude change processes. *J. Exp. Soc. Psychol.* 43 150–156. 10.1016/j.jesp.2006.01.001

[B132] Xu M. Petty R. E. (2021). Two-sided messages promote openness for morally based attitudes. *Pers. Soc. Psychol. Bull.* 48 1151–1166. 10.1177/0146167220988371 33588648

[B133] Yeomans M. Minson J. Collins H. Chen F. Gino F. (2020). Conversational receptiveness: improving engagement with opposing views. *Organ. Behav. Hum. Decis. Process.* 160 131–148. 10.1016/j.obhdp.2020.03.011

[B134] Zimmerman J. M. Coyle V. (2009). *The Way of Council.* Las Vegas, NV: Bramble Books.

